# Leishmanicidal activities of *Artemisia annua* leaf essential oil against Visceral Leishmaniasis

**DOI:** 10.3389/fmicb.2014.00626

**Published:** 2014-11-25

**Authors:** Mohammad Islamuddin, Garima Chouhan, Muzamil Y. Want, Maujiram Tyagi, Malik Z. Abdin, Dinkar Sahal, Farhat Afrin

**Affiliations:** ^1^Parasite Immunology Laboratory, Department of Biotechnology, Jamia Hamdard (Hamdard University)New Delhi, India; ^2^Centre for Transgenic Plant Development, Department of Biotechnology, Jamia Hamdard (Hamdard University)New Delhi, India; ^3^Malaria Group, International Centre for Genetic Engineering and BiotechnologyNew Delhi, India; ^4^Department of Medical Laboratories Technology, Faculty of Applied Medical Sciences, Taibah UniversityMedina, Saudi Arabia

**Keywords:** leishmaniasis, visceral, essential oil, *Artemisia annua*, leishmanicidal, apoptosis, therapeutic efficacy

## Abstract

Visceral leishmaniasis (VL), the second-most dreaded parasitic disease after malaria, is currently endemic in 88 countries. Dramatic increases in the rates of infection, drug resistance, and non-availability of safe vaccines have highlighted the need for identification of novel and inexpensive anti-leishmanial agents from natural sources. In this study, we showed the leishmanicidal effect of essential oil from *Artemisia annua* leaves (AALEO) against *Leishmania donovani in vitro* and *in vivo*. AALEO was extracted by hydrodistillation and characterized by GC-MS, the most abundant compounds were found to be camphor (52.06 %) followed by β-caryophyllene (10.95 %). AALEO exhibited significant leishmanicidal activity against *L. donovani*, with 50 % inhibitory concentration of 14.63 ± 1.49 μg ml^-1^ and 7.3 ± 1.85 μg ml^-1^, respectively, against the promastigotes and intracellular amastigotes. The effect was mediated through programmed cell death as confirmed by externalization of phosphatidylserine, DNA nicking by TdT-mediated dUTP nick-end labeling assay, dyskinetoplastidy, cell cycle arrest at sub-G_0_–G_1_ phase, loss of mitochondrial membrane potential and reactive oxygen species generation in promastigotes and nitric oxide generation in *ex vivo* model. AALEO presented no cytotoxic effects against mammalian macrophages even at 200 μg ml^-1^. Intra-peritoneal administration of AALEO (200 mg/ kg.b.w.) to infected BALB/c mice reduced the parasite burden by almost 90% in the liver and spleen with significant reduction in weight. There was no hepato- or nephro-toxicity as demonstrated by normal levels of serum enzymes. The promising antileishmanial activity shown by camphor-rich AALEO may provide a new lead in the treatment of VL.

## INTRODUCTION

Visceral leishmaniasis (VL), also known as kala azar is a fatal vector-borne illness caused by *Leishmania donovani.* The disease remains a public health problem worldwide causing considerable morbidity and mortality. The chemotherapy of VL has been undermined by drug resistance, variable efficacy, toxicity, parenteral administration, and requirement for long courses of treatment. In India, sodium antimony gluconate (SAG) is no longer useful as a drug because more than 64% of VL patients fail to respond or promptly relapse due to development of resistance in the parasites (Sundar, 2003). Alternative chemotherapeutic measures with amphotericin B (AMB) and its lipid formulations have severe limitations due to their toxic effects and prohibitive high cost. VL has also emerged as an opportunistic infection in individuals carrying HIV infection (Murray, 2004). Thus, there is an urgent need for new and promising compounds for the treatment of this debilitating disease, which mainly affects the rural population.

Essential oils (EO) are volatile mixtures of compounds obtained from spices, aromatic herbs, fruits, and flowers and have been traditionally used in folk medicine to treat several diseases. Various research groups have demonstrated that EO and their main components possess a wide spectrum of biological activities, which may be of great importance in several fields ranging from food chemistry to pharmacology and pharmaceutics. These properties have been attributed to the presence of characteristic constituents, the most abundant being the terpenoids, specially the monoterpenes and sesquiterpenes (Schelz et al., 2010). EO are known to have antibacterial and antifungal properties against human pathogenic microorganisms (Fyfe et al., 1997). EO obtained from *Achillea millefolium* and *Ocimum basilicum* (Santoro et al., 2007) exhibited toxic effects against trypanosomes, the protozoal species closely related to *Leishmania*. A plethora of studies indicate promising leishmanicidal effect of EO from various plants including *Croton cajucara* (Rosa et al., 2003), *Ocimum gratissimum* (Ueda-Nakamura et al., 2006), *Artemisia abyssinica* (Tariko et al., 2010), *A. absinthium* L. (Tariko et al., 2011), and *A. herba-alba* (Mohamed et al., 2010). *A. annua* (Asteraceae), a well-known traditional medicinal plant, has been extensively used as antimalarial and anticancer agent. EO from *A. annua* has been reported to exhibit antifungal, antibacterial (Juteaua et al., 2002) and acaricidal activities (Pirali-Kheirabadi and Teixeira da Silva, 2011).

In this study, we demonstrated the leishmanicidal effect of EO from the leaves of *A. annua* (AALEO) on the promastigotes and amastigotes of *L. donovani* with negligible toxic effect on the host macrophages. We further established the *in vivo* efficacy of AALEO upon intraperitoneal administration to infected BALB/c mice with no impairment of hepatic and renal functions. Our results suggest that AALEO may be a source of novel agents for the treatment of VL.

## MATERIALS AND METHODS

### MATERIALS

Fetal bovine serum (FBS) was procured from Gibco-BRL, DMSO from SRL, methanol from Merck, limulus amebocyte lysate (LAL) kit from Pierce, Thermo Scientific and annexin V-FITC and the Apo- Direct kits from Roche Inc., Basel, Switzerland. All the other chemicals were from Sigma–Aldrich unless otherwise stated.

### PARASITE CULTURE AND MAINTENANCE

*Leishmania donovani* parasites (MHOM/IN/83/AG83) were maintained *in vivo* in BALB/c mice. Promastigotes were routinely cultured at 22°C in M199 medium supplemented with 10% heat inactivated FBS, penicillin (100 IU ml^-1^), streptomycin (100 μg ml^-1^). Log phase promastigotes were sub-cultured every 72–96 h, the inoculum being 2 × 10^6^ cells ml^-1^.

### CELL CULTURE

Cell line, RAW 264.7 were grown at 37°C in RPMI-1640 medium (pH 7.4) supplemented with 10% heat-inactivated FBS for 48–72 h in a humidified atmosphere of 5% CO_2_ and sub-cultured in fresh RPMI-1640 medium at an average density 2 × 10^5^ cells ml^-1^.

### ANIMALS

Female BALB/c mice, aged 6–8 weeks and weighing about 20–25 g were used in the present study. All the animals were individually housed in standard size polycarbonate cages with controlled conditions of temperature (23 ± 1°C), humidity (55 ± 10%), 12:12 h of light and dark cycles and fed with a standard pellet diet (Ashirwad Industries, Chandigarh, India) and filtered water *ad libitum* in the Central Animal House of Jamia Hamdard according to the internationally accepted principles. *A prior* approval (Ethical approval judgment number 459) was obtained from the Jamia Hamdard Animal Ethics Committee (JHAEC) for the study protocol. JHAEC is registered under the Committee for the purpose of control and supervision of experiments on animals (CPCSEA).

### PLANT MATERIAL AND THE ESSENTIAL OIL

Fresh *A. annua* leaves were collected from the Herbal Garden of Jamia Hamdard. The essential oil was extracted with a modified Clevenger-type apparatus (Borosilicate) by steam distillation (Tzenkova et al., 2010). Briefly, 1 kg of the *A. annua* fresh leaves were boiled in 3 L of distilled water for 4 h. At the end of this procedure, the yield obtained was 0.6% of oil on a dry weight basis (4.2 g of *A. annua* leaves). After extraction, the essential oil obtained was carefully separated, transferred to an opaque glass vial with rubber lid and covered with aluminum foil to protect from light. The EO was stored in a refrigerator (–20°C) prior to analysis. For biological assays, EO was reconstituted aseptically to 1 mg ml^-1^ in DMSO (cell culture grade) and diluted further in culture medium to achieve a final DMSO concentration of not more than 0.2%.

### GC AND GC-MS ANALYSIS OF THE ESSENTIAL OIL FROM *A. annua* LEAVES

The chemical composition and characterization of the bio-active compounds from the essential oil of AALEO was analyzed by gas chromatography (GC) and GC-mass spectrometry (GC-MS) using a SHIMADZU QP2010 with a RTX5 MS column (30 m, film 0.25 μm, ID 0.25 mm). The temperature of the column was programmed from 45 to 270°C at 5°C min^-1^, the injector and detector temperatures for the analysis were 250°C. Helium was used as the carrier gas at a flow rate of 1.5 ml min^-1^. The identification of chemical constituents was performed on the basis of retention indices (RI) determined with reference to a homologous series of n-alkanes, under identical experimental conditions, co-injected with standards (RESTEK, 110 Benner Circle, Bellefonte, PA, USA), followed by MS library search (WILEY8.LIB and NIST08.LIB Version; Mdoe et al., 2014).

### PROMASTIGOTE PROLIFERATION KINETICS AND GROWTH REVERSIBILITY ASSAY

Promastigotes (2 × 10^6^ cells ml^-1^) in M199 medium were incubated at 22°C with AALEO (100 μg ml^-1^) in M199 containing 10% FBS. Pentamidine at the same concentration served as the reference antileishmanial drug while 0.2% DMSO, which represented the highest concentration in the test oil was used as solvent control. Parasites in media alone were taken as control. Promastigotes were enumerated daily for 7 days in a hemocytometer under phase contrast microscope using 40X objective (Islamuddin et al., 2014). Treated and untreated parasites after 7 days in culture, were washed and re-suspended in fresh medium, and allowed to grow for a further 4 days at 22°C. The viability of the parasites was determined microscopically to affirm the leishmanicidal effect of AALEO (Afrin et al., 2001).

### DETERMINATION OF PROMASTIGOTE CELLULAR MORPHOLOGY

Changes in the cellular morphology of parasites as a result of AALEO treatment was observed microscopically. Briefly, the promastigotes (2 × 10^6^ cells ml^-1^) were incubated at 22°C in the absence or presence of AALEO and pentamidine for 72 h at a concentration of 100 μg ml^-1^ and observed under 40X objective using a phase-contrast microscope. At least 20 microscopic fields were examined for each sample. Data were recorded using NIS-Elements imaging software (Paris et al., 2004).

### DOSE-DEPENDENT EVALUATION OF ANTI-PROMASTIGOTE ACTIVITY AND DETERMINATION OF IC_50_

Promastigotes at a density of 2 × 10^6^ cells ml^-1^ were incubated in the absence and or presence of AALEO at serial three fold dilutions starting at 100 μg ml^-1^ (100, 33.33, 11.11, 3.70, 1.23, and 0.41 μg ml^-1^) for 72 h at 22°C. Pentamidine was used as a standard antileishmanial drug. The mean percentage viability was calculated as follows: (Mean cell number of treated parasites/Mean cell number of untreated parasites) X 100. The 50 % inhibitory concentration (IC_50_) i.e., the concentration that decreased the cell growth by 50 % was determined by graphical extrapolation after plotting the graph of percent (%) viability versus concentration of the drug (Islamuddin et al., 2014).

### ANTI-AMASTIGOTE ACTIVITY AND NITRITE PRODUCTION IN AN *EX-VIVO* MACROPHAGE–MODEL

Macrophages from the RAW 264.7 cell line (1 × 10^6^ cells ml^-1^) were allowed to adhere to round glass cover slips in a 24-well tissue culture plate and incubated for 12 h at 37°C in a carbon dioxide incubator (5% CO_2_). After removal of the non-adherent macrophages, the cells were infected with stationary phase promastigotes using a cell to parasite ratio of 1:10 and incubated at 37°C for 24 h. The non-phagocytosed parasites were removed by washing, and the infected macrophages incubated with AALEO (0–100 μg ml^-1^) for an additional 48 h. The cover slips were fixed in methanol and giemsa-stained for microscopic evaluation (100X) of amastigote infectivity. At least 200 macrophages per cover slip were analyzed, and the concentration that decreased amastigotes by 50% (IC_50_) was determined (Islamuddin et al., 2014).

The supernatant from control *L. donovani* infected and treated macrophages (RAW 264.7) were analyzed for nitrite content by the Griess reaction. In brief, equal volume of Griess reagent [1% sulphanilamide and 0.1% *N*-(1-naphthyl) ethylenediamine dihydrochloride in 5% phosphoric acid] was added to the culture supernatant and the plates were incubated for 10 min at room temperature using sodium nitrite as a standard. The absorbance at 550 nm was measured, and the concentration of nitrite was calculated by a linear regression analysis using the standard curve generated with sodium nitrite (Varela et al., 2012). All the reagents including AALEO, were free of lipopolysaccharide (0.2 ng ml^-1^ endotoxin) as determined by LAL chromogenic endotoxin quantitation kit as per the manufacturer’s instructions (Pierce, Thermo Scientific).

### *EX VIVO* CYTOTOXICITY ASSAY

To evaluate the adverse toxicity of AALEO on mammalian cells, the murine macrophage cell line (RAW 264.7) cultured in RPMI 1640 medium were exposed to increasing concentrations of AALEO (0–200 μg ml^-1^) at 37°C, 5 % CO_2_ for 48 h. Pentamidine as the reference drug, and macrophages without any treatment were taken as control. The percentage of cell viability was evaluated by MTT (3-{4,5-dimethylthiazol-2-yl}-2,5-diphenyltetrazolium bromide) assay (Das et al., 2008).

### ANALYSIS OF PHOSPHATIDYLSERINE (PS) EXTERNALIZATION

To visualize PS exposure, double staining with Annexin-V and PI was performed with minor modifications. Briefly, promastigotes (2 × 10^6^ cells ml^-1^) were incubated with AALEO at a concentration of 100 μg ml^-1^ for 72 h. Untreated and treated parasites were centrifuged at 3000 × *g* for 10 min, washed twice in cold PBS and the pellet was resuspended in Annexin V-FLUOS labeling solution (100 μl, containing both Annexin-V and PI), as per the manufacturer’s instructions (Roche). After 15 min of incubation in the dark at 26°C, 400 μl of incubation buffer was added, mixed and acquired using a BD LSR II flow cytometer and analyzed using Deva software. Pentamidine served as the reference antileishmanial drug (100 μg ml^-1^) while parasites without any treatment were taken as control.

### *IN SITU* DETECTION OF DNA FRAGMENTATION BY TERMINAL DEOXYNUCLEOTIDYL TRANSFERASE (TdT) MEDIATED dUTP NICK-END LABELING (TUNEL) ASSAY

*In situ* detection of DNA fragments within the promastigotes was analyzed by TdT-mediated dUTP nick-end labeling (TUNEL) assay using an *in situ* cell death detection kit (Roche) according to the manufacturer’s instructions. Briefly, promastigotes (2 × 10^6^ cells ml^-1^) were incubated at 22°C with AALEO (100 μg ml^-1^), pentamidine (100 μg ml^-1^) as the reference drug and with media alone as control. After 72 h, the cells were washed, fixed with 4% paraformaldehyde on ice for 1 h, washed with PBS and incubated with 3% H_2_O_2_ in methanol for 10 min at 25°C. This was followed by washing with PBS and the cells were then permeabilized with freshly prepared chilled 0.1% Triton X-100 for 5 min on ice. The cells were washed twice with PBS, after which 50 μl of reaction mixture containing TdT and FLUOS labeled dUTP was added for 1 h at 37°C, washed and finally resuspended in PBS for acquisition in a BD LSR II flow cytometer and analyzed using Deva software.

In a parallel experiment, the stained samples were visualized under a high-resolution fluorescence microscope under 10× objective, and the images captured and processed.

### STUDY OF DYSKINETOPLASTIDY BY FLUORESCENCE MICROSCOPY

To detect the changes in kinetoplast DNA (kDNA) of promastigotes, untreated and AALEO (100 μg ml^-1^) treated exponential-phase promastigotes (2 × 10^6^ cells ml^-1^) after 72 h were washed twice with PBS, fixed with 80% chilled ethanol and kept at 4°C for 24 h. The cells were then washed with PBS and the pellet was resuspended in 500 μl DNase-free RNase (200 μg ml^-1^) for 1 h at 37°C. The cells were stained with PI (50 μg ml^-1^) and incubated in the dark for 20 min at 4°C. An aliquot of 20 μl was taken from each sample, placed on a glass slide and observed directly under 20× objective (without fixation) under a high-resolution fluorescence microscope (Nikon). The images were processed using NIS-Elements imaging software. At least 20 microscopic fields were observed for each sample (Singh et al., 2009).

### EFFECT OF AALEO ON CELL CYCLE

Percentage of cells in the different phases of cell cycle was detected through flow cytometry by propidium iodide (PI) staining. Briefly, promastigotes (2 × 10^6^cells ml^-1^) were treated with AALEO (100 μg ml^-1^) for 72 h at 22°C, washed twice with PBS, fixed with 80% chilled ethanol and kept at 4°C for 24 h. The cells were then washed with PBS and the pellet resuspended in 500 μl DNase free RNase A (200 μg ml^-1^) for 1 h at 37°C. The cells were stained with PI (50 μg ml^-1^) and incubated in the dark for 20 min at 4°C. Data acquisition was carried out using BD FACS Calibur flow cytometer and analyzed using Cell Quest software (Queiroz et al., 2014).

### MITOCHONDRIAL MEMBRANE POTENTIAL (ΔΨm) DETERMINATION

The mitochondrial transmembrane potential (ΔΨm) was measured using a fluorogenic cell-permeable cationic dye, JC-1 (5,59,6,69-tetrachloro-1,19,3,39-tetraethylbenzimidazolylcarbo-cyanine iodide). To analyse the changes in ΔΨm, the promastigotes after exposure to AALEO (100 μg ml^-1^) for 72 h were centrifuged, resuspended in PBS containing JC-1 (10 μg ml^-1^) and incubated at 37°C for 10 min. The cells were then analyzed by flow cytometry, BD LSR II and DEVA software. The ratio of the red/green fluorescence intensities was considered as the relative ΔΨm value (Roy et al., 2008).

### ESTIMATION OF INTRACELLULAR REACTIVE OXYGEN SPECIES (ROS) LEVELS

To monitor the effect of AALEO on the generation of reactive oxygen species (ROS), late log phase promastigotes were treated with AALEO at 22°C for 72 h. The cells were washed twice with PBS and finally resuspended in 500 μl PBS, loaded with a cell permeant probe, 2, 7 dichlorodihydrofluorescein diacetate (H_2_DCFDA; 10 μM) and incubated for 30 min at 25°C. The cells were analyzed for intracellular ROS by BD LSR II flow cytometer and DEVA software (Misra et al., 2009).

### *IN VIVO* EFFICACY OF AALEO AGAINST *L. donovani*

Female BALB/c mice (6 to 8-weeks old) were infected with stationary phase promastigotes (2 × 10^7^ per animal) through tail vein. Two months post infection, parasite burden was confirmed in two arbitrarily selected animals; after which, the mice were randomly assigned into four groups. Group-A comprised of control, infected mice without treatment (INF), vehicle control Group-B received normal saline (VC). Group-C was administered AALEO intraperitoneally (i.p.) and further divided into three subgroups (*n* = 5) that received three doses of AALEO (50, 100, 200 mg/kg body weight {b.w.}) for ten consecutive days. Group-D received the standard antileishmanial drug, AMB (5 mg/kg b.w. for 10 alternate days, intravenously {i.v.}) and served as the positive control. Ten days post treatment, mice were euthanized by CO_2_ asphyxiation, the liver and spleen parasite burdens were determined from giemsa-stained multiple impression smears and expressed as Leishman-Donovan units (LDU) that was calculated as the number of parasites per 500 nucleated cells × organ weight (mg) (Dutta et al., 2008).

The percent reduction in parasite load (% protection) was calculated using the formula.

$$\%\mathrm{Pr}otection=\frac{LDU\,\;\;\;of\,\;\;\inf ected\,\;\;control-LDU\,\;\;of\,\;\;treated\,\;\;animals}{LDU\,\;\;of\,\;\;\inf ected\,\;\;control}\times 100$$

Cure or protection correlated with a fall in hepatosplenomegaly and elimination of parasites to negligible levels.

### *IN VIVO* TOXICITY ASSAY

Hepatic and renal functions of BALB/c mice were evaluated in treated and untreated animals as described previously (Agarwal et al., 2012). Two-weeks post treatment, blood was drawn from the retro-orbital plexus and serum was separated by centrifugation at 5000 × *g* for 2–3 min. The serum was stored at -80°C until use. The hepatic and renal functions were assessed by measuring the levels of serum glutamic oxaloacetic transaminase (SGOT), serum glutamic pyruvic transaminase (SGPT), and alkaline phosphatase (ALP), urea and creatinine using commercially available kits (Span Diagnostics Ltd.). The hepatic and renal functions were similarly evaluated in normal BALB/c mice at higher (200 mg/kg b.w.) dose of AALEO.

### STATISTICAL ANALYSIS

The *in vitro* experiments were performed at least in triplicate. A minimum of five mice per group was used for all *in vivo* experiments. Error bars represent the SEM.

The statistical significance of differences between the groups was determined as described in the figure legends using ANOVA followed by Tukey’s test by Graph Pad Prism 5 software, and a *p-*value of <0.05 was considered statistically significant.

## RESULTS

### GC AND GC-MS ANALYSIS OF AALEO

The composition of the essential oil exhibiting leishmanicidal activity was studied by GC and GC-MS. The major chemical constituent was identified as Camphor; 52.06% (**Table 1**); by comparison of mass spectral data, retention times (RT), and RI. The other constituents present in the essential oil were β-caryophyllene (10.95 %), 1,8-cineole (5.57 %), β-caryophyllene oxide (4.21 %), β-farnesene (3.83 %), α-copaene (2.91 %), β-cyperone (1.93 %), α-selinene (1.54 %), and *trans*-pinocarveol (1.22 %) as presented in **Table 1**.

**Table 1:** Phytochemical fingerprinting of AALEO by GC-MS.

Peak	Retention time (RT)	Retention index (RI)	Area	Area%	Compound
1	3.250	876	73280	0.50	β-Pinene
2	4.549	929	810646	5.57	1,8-Cineole
3	5.586	972	167764	1.15	o-Cymol
4	5.683	976	38352	0.26	Isovaleric acid
5	9.449	1093	47296	0.33	1-Octen-3-ol
6	9.990	1108	41392	0.28	α-Cubebene
7	11.028	1133	422708	2.91	a-Copaene
**8**	**11.672**	1149	**7573773**	**52.06**	**Camphor**
9	12.269	1164	47711	0.33	3-Caren-10-al
**10**	**13.772**	1201	**1593527**	**10.95**	**β -Caryophyllene**
11	14.451	1217	38244	0.26	δ-Myrtenal
12	15.073	1232	177125	1.22	(E)-Pinocarveol
13	15.308	1238	54947	0.38	Germacrene B
14	15.540	1243	556838	3.83	β-Farnesene
15	15.784	1249	224008	1.54	α-Selinene
16	16.035	1255	49450	0.34	β-Chamigrene
17	16.119	1257	65761	0.45	g-Muurolene
18	16.248	1260	50529	0.35	α-Terpineol
19	16.330	1262	80643	0.55	Borneol
20	16.690	1270	165303	1.14	α-Amorphene
21	16.965	1277	280737	1.93	β-Cyperone
22	17.298	1285	41837	0.29	S (+)-Carvone
23	17.945	1300	78707	0.54	δ-Cadinene
24	18.358	1310	25951	0.18	(E)-Carveol
25	18.636	1316	32965	0.23	1,3-Dimethyladamantane
26	18.761	1319	44074	0.30	Myrtenol
27	19.907	1347	75486	0.52	(Z)-Carveol
28	20.285	1356	18366	0.13	*p*-Cymen-8-ol
29	20.807	1368	41234	0.28	Benzyl valerate
30	21.408	1383	49028	0.34	(2E)-2-Methyl-4-(2,6,6-trimethyl-1-cyclohexen-1-yl)-2-butenal
31	22.335	1405	20933	0.14	Vulgarone B
32	23.518	1434	49521	0.34	Viridiflorol
**33**	**23.791**	1441	**611878**	**4.21**	β **-Caryophyllene oxide**
34	24.383	1455	54592	0.38	Ethyl chrysanthemate
35	24.989	1470	107844	0.74	Isoaromadendrene epoxide
36	26.576	1510	78911	0.54	Cubenol
37	27.128	1524	143531	0.99	Spathulenol
38	28.175	1552	34667	0.24	β-Acoradiene
39	28.495	1560	28088	0.19	(-)-Globulol
40	28.822	1568	127617	0.88	Longifolenaldehyde
41	29.291	1580	258844	1.78	Diepicedren-1-oxide
42	43.679	1972	63369	0.44	β-Panasinsene
			14547477	100.00	

*Retention index as determined on a RTX5 MS column using the homologous series of *n*-alkanes. The major constituents are represented in bold letters.*

### AALEO MEDIATES DEATH IN PROMASTIGOTES

**FIGURE 1:**
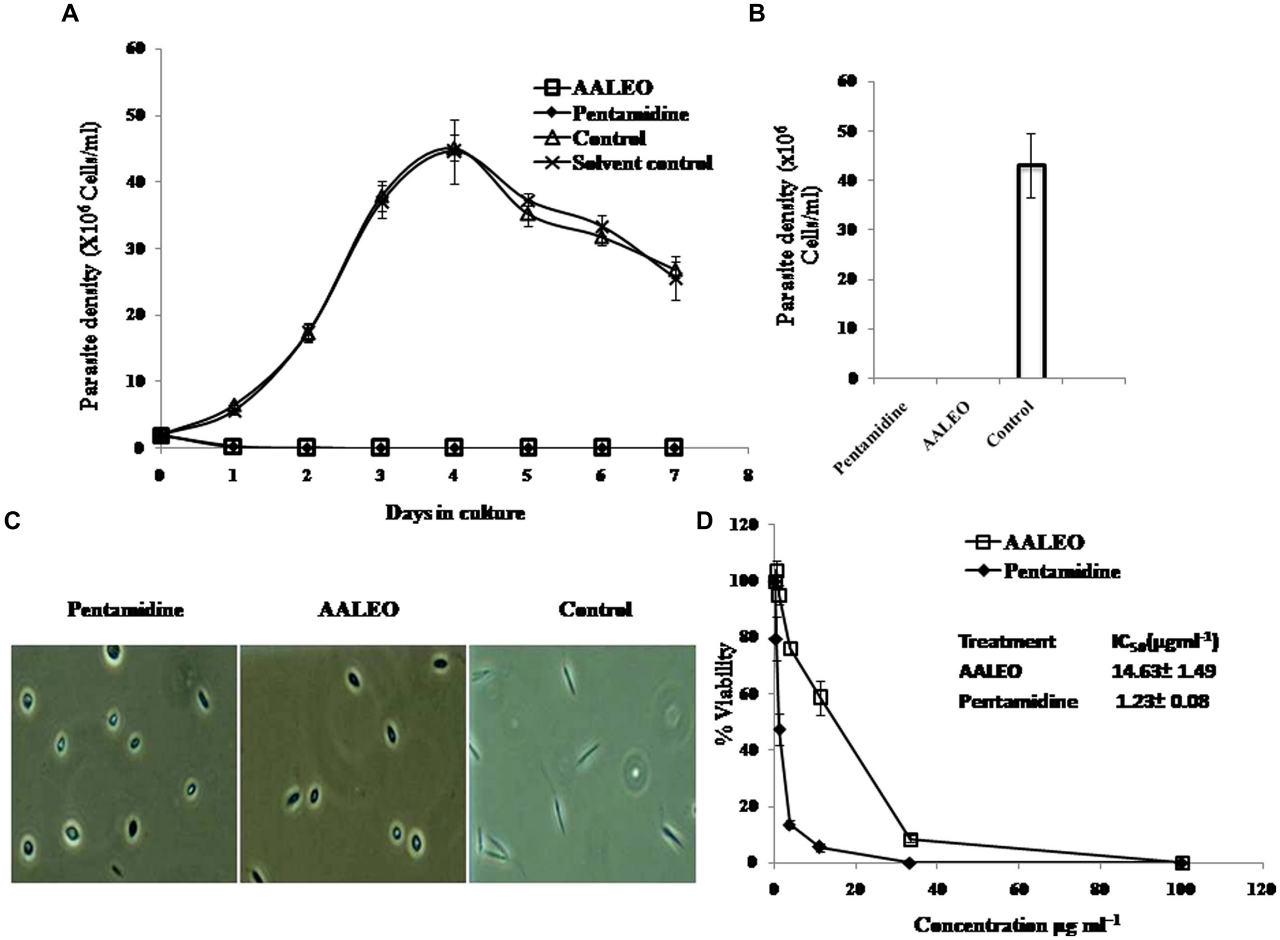
**Anti-promastigote activity of *Artemisia annua* leaves (AALEO).** Log phase *Leishmania donovani* promastigotes were incubated with AALEO (100 μg ml^-1^) for 7 days at 22°C as described in “Materials and Methods.” **(A)** Viable parasites enumerated microscopically every day **(B)** promastigote growth reversibility analysis after 4 days of culture, post AALEO withdrawal **(C)** morphological analysis of promastigotes by light microscopy (40×) at 72 h post-treatment and **(D)**% viability ascertained upon incubation of promastigotes for 72 h with increasing concentrations of AALEO (0–100 μg ml^-1^) for IC_50_ determination (inset). Each point or bar corresponds to the mean ± SE of triplicate samples and is representative of one of three independent experiments.

The anti-proliferative effect of AALEO on promastigotes was studied by monitoring the kinetics of cell growth. AALEO exhibited a time-dependent inhibition in the rate of cell proliferation at a concentration of 100 μg ml^-1^. All the cells appeared dead after 72 h of incubation (**Figure 1A**). Pentamidine used as a positive control, showed a similar trend in time-dependent parasite killing. Untreated promastigotes (without any drug) proliferated at a normal rate. Solvent control (0.2% DMSO) showed no adverse effect on the promastigote viability.

### LEISHMANICIDAL ASSAY OF THE TREATED PROMASTIGOTES

To corroborate the lethal effects of AALEO on promastigotes, treated and untreated parasites after 4 days were washed and re-suspended in fresh media and their viability was determined under phase contrast microscope. After an additional 4 days in culture, the parasites did not revert back to their normal morphology and still appeared dead in case of prior incubation with AALEO, as also observed in positive control (pentamidine), confirming their leishmanicidal effects. Untreated control promastigotes reverted to late log phase (**Figure 1B**).

### AALEO CAUSES ALTERATIONS IN CELLULAR MORPHOLOGY OF PROMASTIGOTES

Microscopic inspection of promastigotes upon treatment with AALEO (100 μg ml^-1^) revealed that the parasites altered to an ovoid shape with cell shrinkage, loss of flagella, cytoplasmic condensation, resulting in complete circularization of the promastigotes and substantial reduction in size compared to the untreated control. These changes, typical of programmed cell death (PCD; **Figure 1C**), were also observed in pentamidine treated parasites.

### EVALUATION OF IC_50_ OF AALEO AGAINST *L. donovani* PROMASTIGOTES

*Artemisia annua* leaves (0–100 μg ml^-1^) treatment demonstrated a dose-dependent killing of the promastigotes and a 50% inhibitory concentration was achieved at 14.63 ± 1.49 μg ml^-1^ (**Figure 1D**). The established antileishmanial drug pentamidine, used as a positive control, showed a similar trend in parasite killing. Parasite viability was not affected by DMSO (0.2%, data not shown) used as solvent control.

### ANTI-AMASTIGOTE ACTIVITY OF AALEO

**FIGURE 2:**
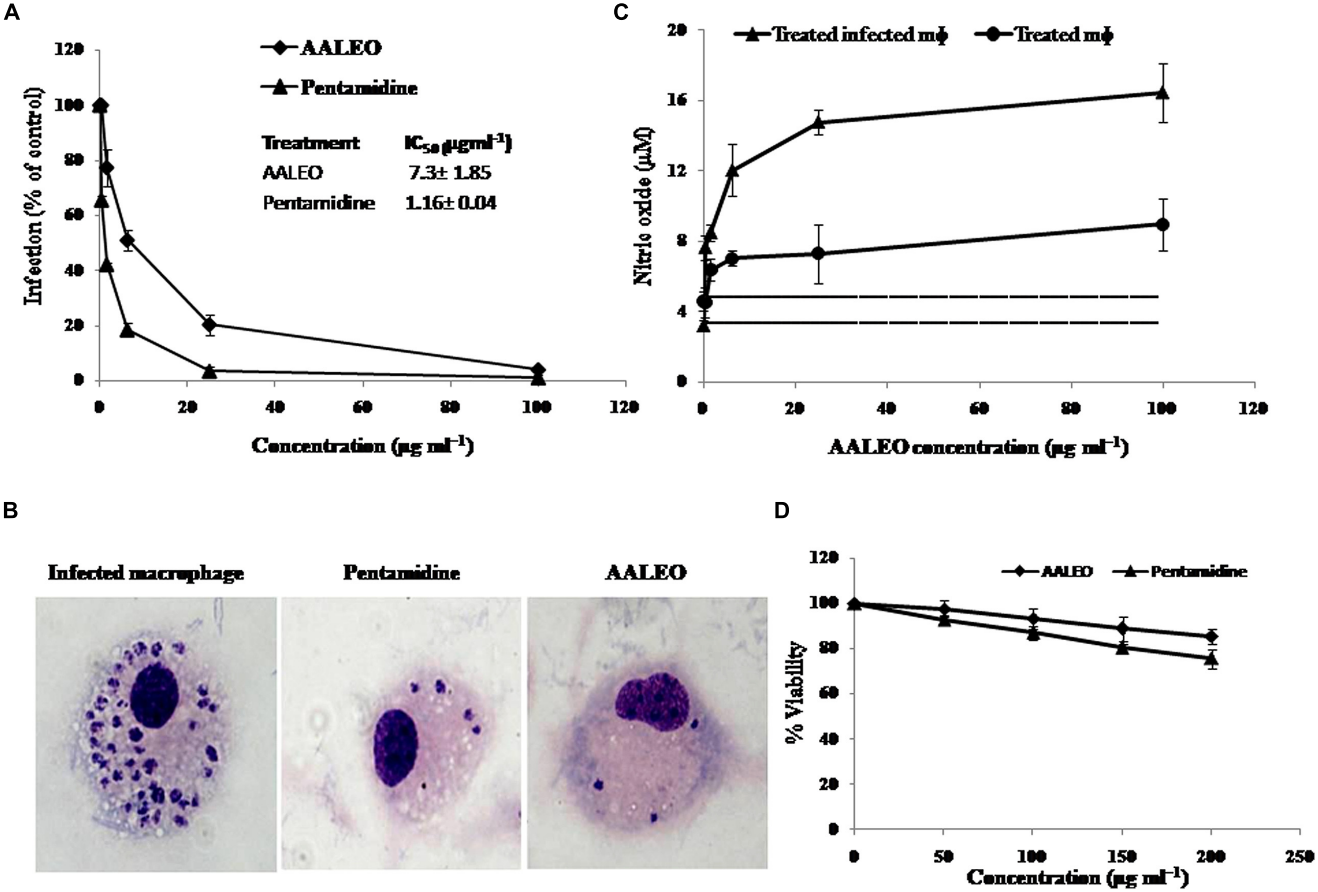
**Effect of AALEO on *L. donovani* intracellular amastigote forms.** Promastigote-infested RAW 264.7 macrophages were incubated at 37°C in a CO_2_ incubator with serial fourfold dilutions of AALEO (starting at 100 μg ml^-1^) for 48 h. **(A)**% infectivity estimated for determination of IC_50_ on amastigotes (inset) **(B)** Micrographs of giemsa-stained infected macrophages upon treatment (100×) **(C)** NO generation by uninfected (filled circle) or infected (filled triangle) macrophages post-incubation with AALEO. The dotted and bold dotted lines represent the basal levels of NO produced by uninfected and infected macrophages, respectively. **(D)** Cytotoxicity of AALEO to RAW 264.7 macrophages ascertained as % viability 48 h post-incubation with increasing concentrations of AALEO or pentamidine (0–200 mg ml^-1^). Each point corresponds to the mean ± SE of triplicate samples and is representative of one of three independent experiments.

The activity of AALEO on phagocytosed amastigotes within the macrophages was analyzed by microscopic observation of giemsa-stained cells. Macrophages entry by promastigotes involves the formation of membrane bound parasitophorous vacuoles, where they transform into non-motile amastigote form. The parasites survival within the parasitophorous vacuoles determines the pathogenesis. Therefore, it was important to test the activity of AALEO toward the macrophage-resident amastigotes. Treatment with AALEO (0–100 μg ml^-1^) demonstrated a dose-dependent inhibition of amastigote infectivity with IC_50_ of 7.3 ± 1.85 μg ml^-1^ (**Figure 2A**). Giemsa-stained micrographs of infected macrophages showed almost total clearance of the intracellular amastigotes at the highest dose (100 μg ml^-1^); similar effect was observed with pentamidine (**Figure 2B**).

### AALEO INDUCES NITRIC OXIDE PRODUCTION IN *EX VIVO* MODEL

Estimation of nitric oxide (NO) in the culture supernatants of murine non-parasitized and parasitized macrophages (RAW 264.7) indicated low basal levels of NO in the parasitized macrophages corroborating with disease progression. Following addition of AALEO (0–100 μg ml^-1^) for 48 h, a dose-dependent increase in NO production occurred in the infected macrophages (**Figure 2C**) that was distinctly higher than that released by normal macrophages. AALEO at 100 μg ml^-1^ induced 8.96 ± 1.45 and 16.43 ± 1.68 μM of NO in normal and infected macrophages, respectively.

### CYTOTOXICITY OF AALEO ON RAW 264.7 MACROPHAGES CELL LINE

*In vitro* cytotoxicity assay was done with the murine macrophage cell line RAW 264.7 to check the adverse effects of AALEO, using pentamidine as a reference drug. The toxicity assay revealed that AALEO had no adverse effects on the viability and morphology of the macrophages (**Figure 2D**).

### AALEO INDUCES PS EXTERNALIZATION IN *L. donovani* PROMASTIGOTES

**FIGURE 3:**
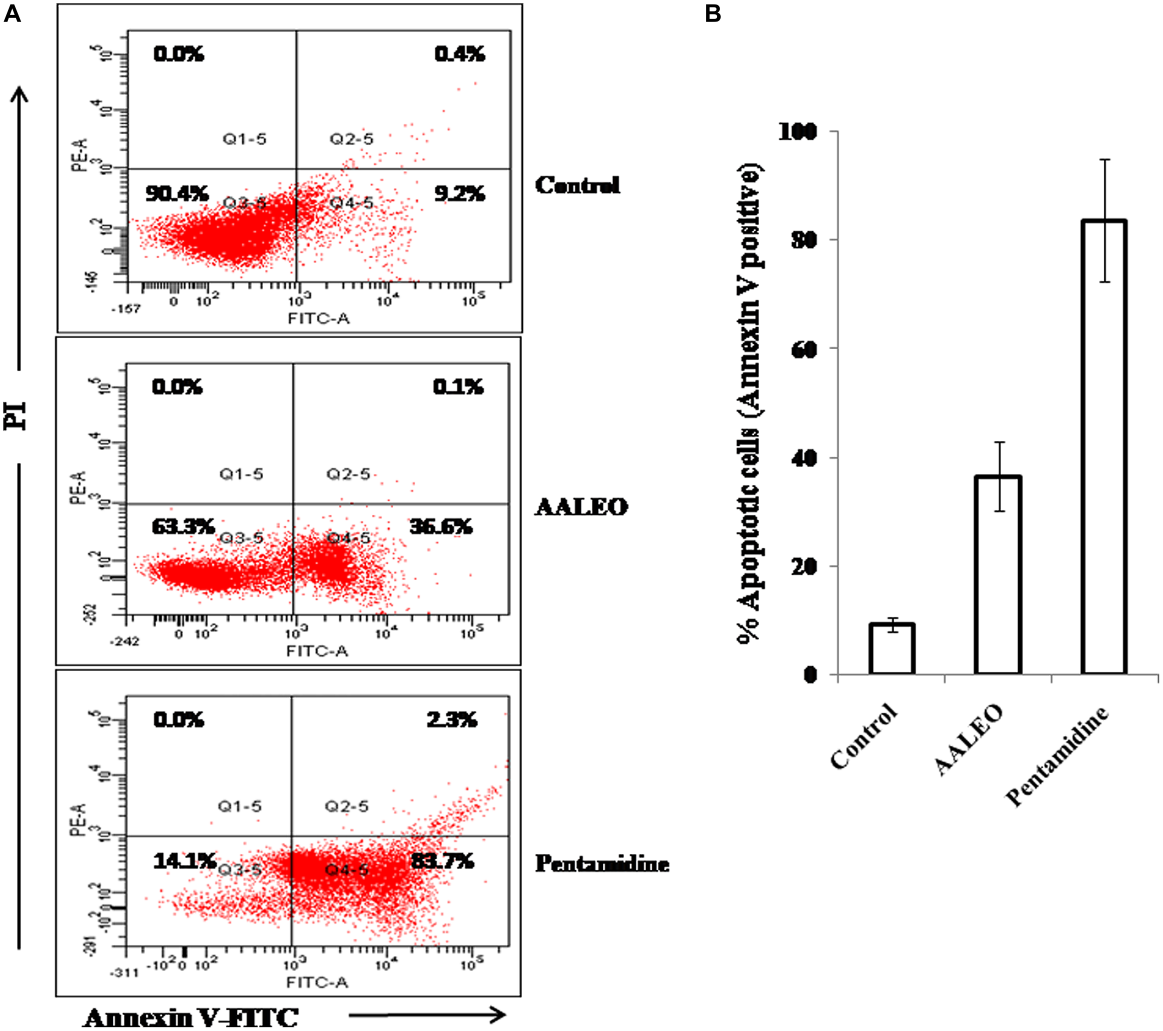
**Detection of apoptosis in *L. donovani* promastigotes by annexin V-FITC and PI co-staining.** Parasites were incubated with AALEO or pentamidine (100 μg ml^-1^) for 72 h, and analyzed by flow cytometry as described in the Methods. **(A)** Dual parametric dot plots combining Annexin V-FITC and PI fluorescence, **(B)** % apoptotic cell population. This is a representative profile of three independent experiments.

During apoptosis in unicellular parasitic cells, PS is translocated from the inner side to the outer leaflet of the plasma membrane. Annexin V, a Ca^2+^-dependent phospholipid binding protein with an affinity for PS, is routinely used to label externalization of PS. Since annexin V can also label necrotic cells, PI was used to differentiate among apoptotic cells (annexin V +ve and PI –ve), necrotic cells (annexin V –ve and PI +ve), late apoptotic cells (annexin V +ve and PI +ve) and live cells (annexin V –ve and PI –ve). In control late log phase promastigotes, the basal binding of annexin V was 9.2%. Whereas, with AALEO, the annexin V binding (a correlate of apoptotic cells) increased to 36.6% and, to 83.7% with pentamidine (**Figures 3A,B**). Taken together, our data indicate that AALEO caused apoptosis in promastigotes with externalization of PS.

### OLIGONUCLEOSOMAL DNA FRAGMENTATION IN AALEO TREATED PROMASTIGOTES

**FIGURE 4:**
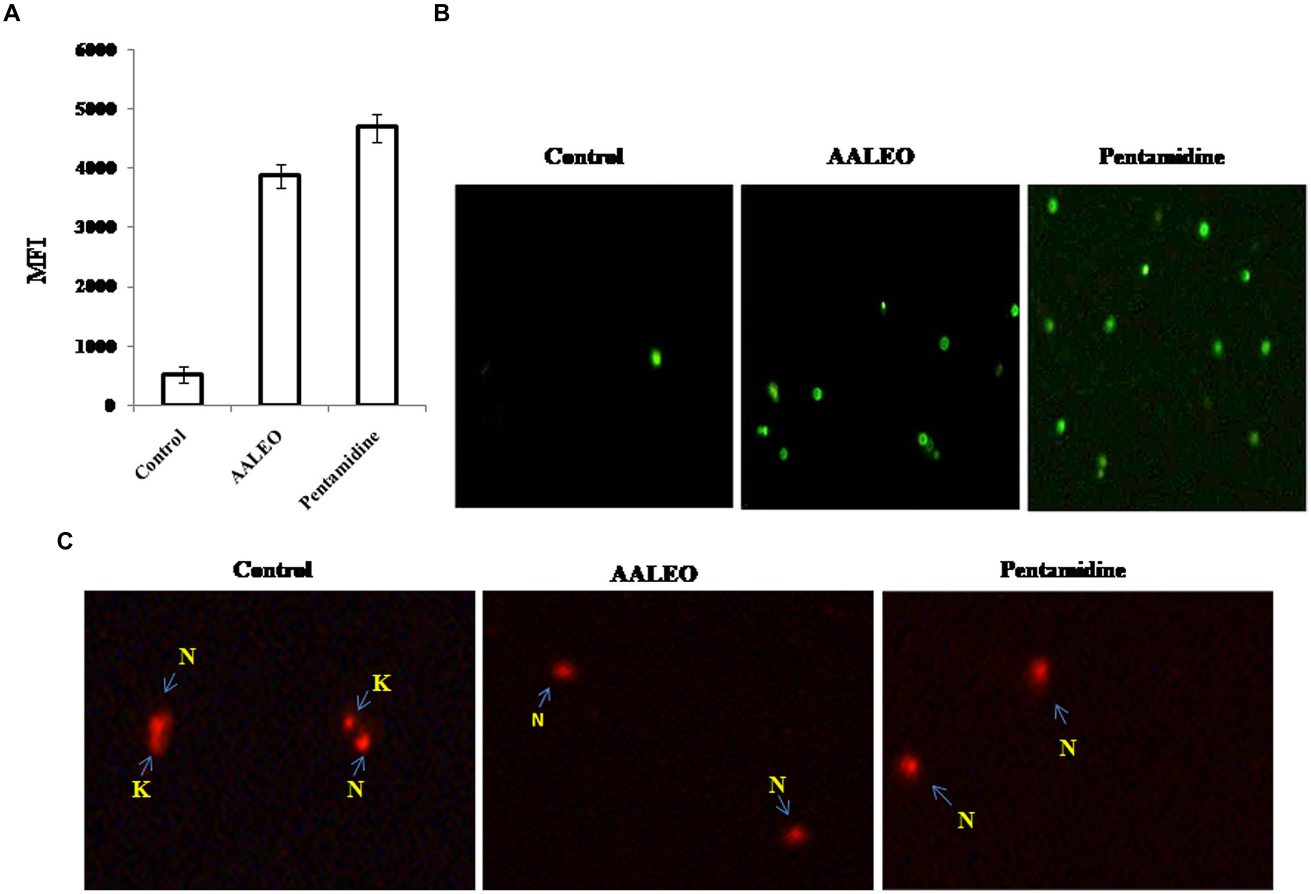
***Artemisia annua* leaves treated promastigotes represent TdT-mediated dUTP nick-end labeling (TUNEL) positivity and dyskinetoplastidy.** Exponential phase promastigotes were incubated with AALEO or pentamidine (100 μg ml^-1^) for 72 h. Cells were fixed and stained with dUTP–FITC in the presence of TdT. **(A)** Bar graphs representing MFI of TUNEL expression as analyzed by flow cytometry **(B)** Image acquisition via a fluorescence microscope under 10× objective. **(C)** Treated and untreated parasites were probed with PI for assessment of dyskinetoplastidy and images taken under 20× objective as described in Section “Materials and Methods.” N and K represent nuclear and kinetoplast DNA, respectively. Each bar corresponds to the mean ± SE of triplicate samples and is a representative profile of three independent experiments. Images are representative of one of three similar results.

One of the important biochemical hallmarks of eukaryotic apoptotic cell death is the fragmentation of nuclear DNA into nucleosomal units. DNA nicking following treatment with AALEO was detected using a TUNEL assay in which the proportion of DNA nicks was quantified by measuring the binding of dUTP–FLUOS to the nicked ends via TdT. Thus, the proportion of DNA nicks was directly proportional to the fluorescence obtained from dUTP–FLUOS. Promastigotes treated with 100 μg ml^-1^ of AALEO for 72 h showed an increase in nuclear DNA fragmentation as evidenced from dUTP–FLUOS binding. The mean fluorescence intensity (MFI) of untreated promastigotes (522) increased to 4694 and 3876 upon treatment with AALEO and pentamidine, respectively (**Figure 4A**). The results clearly indicate that AALEO caused DNA nicking in promastigotes.

Fluorescence microscopic observation showed the appearance of green fluorescence in treated samples, representing incorporation of dUTP labeled with FLUOS (**Figure 4B**). Thus, the results further confirm AALEO-mediated induction of apoptosis in *L. donovani* promastigotes.

### AALEO-INDUCED CYTOTOXICITY CAUSES DYSKINETOPLASTIDY IN PROMASTIGOTES

The kDNA has been shown to be susceptible to elimination (dyskinetoplastidy) by certain DNA intercalating drugs for example, acriXavine and berenil. We observed that AALEO induced genomic DNA fragmentation in *L. donovani* promastigotes. Based on this, we investigated whether AALEO caused any damage to kDNA. Untreated and promastigotes treated with AALEO (100 μg ml^-1^) were permeabilized and stained with PI that binds with DNA of the promastigotes, i.e., kDNA and nuclear DNA. The loss of mitochondrial kDNA was detected at 72 h of treatment with approximately all the cells showing dyskinetoplastic feature (**Figure 4C**) against the intact kDNA structure in untreated cells.

### AALEO-INDUCED APOPTOSIS CAUSES CELL-CYCLE ARREST IN *L. donovani* PROMASTIGOTES

**FIGURE 5:**
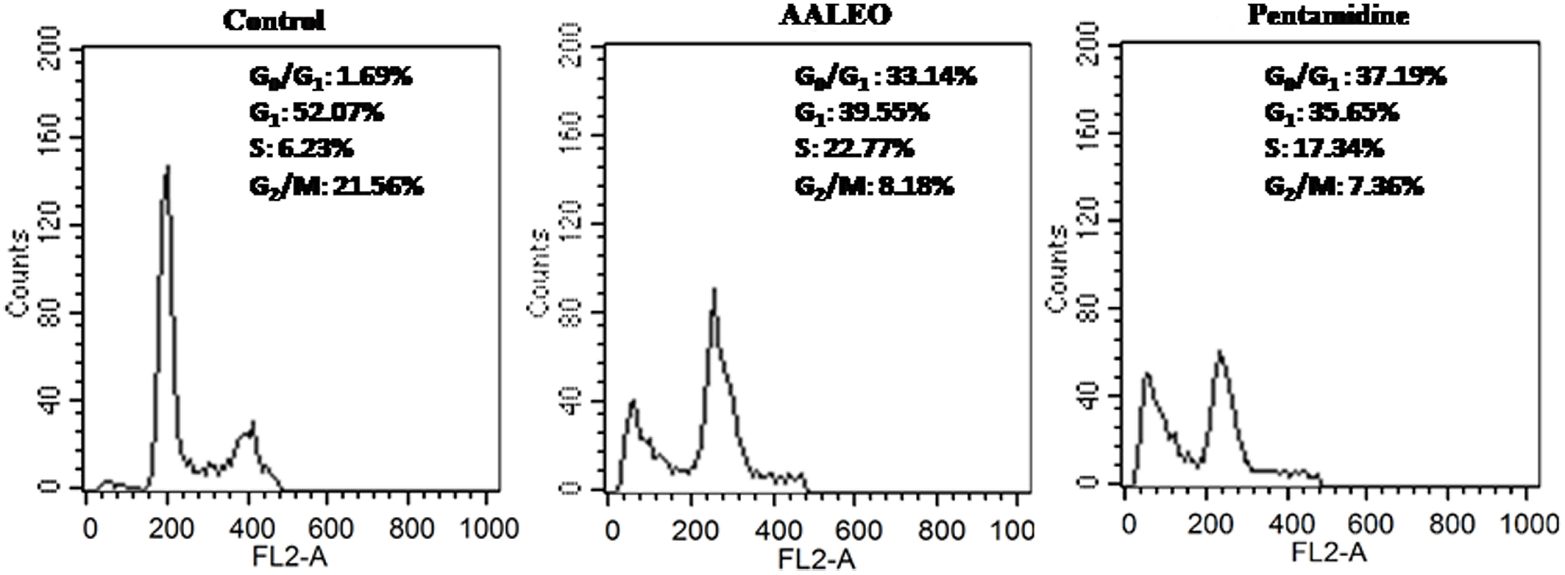
***Artemisia annua* leaves induces cell cycle arrest in *L. donovani* promastigotes at sub-G_**0**_–G_**1**_ phase.** After treatment with AALEO and pentamidine (100 μg ml^-1^) for 72 h, the promastigotes were fixed in chilled methanol, probed with PI and the cells acquired using a BD LSR-II flow cytometer for subsequent analysis using Cell Quest software as described in Section “Materials and Methods.” Inset shows the cell cycle distribution. This is the representative profile of three independent experiments.

The changes in cell cycle progression in *L. donovani* promastigotes were examined by flow cytometric analysis of permeabilized and PI stained cells after treatment with AALEO (100 μg ml^-1^) for 72 h. In a given cell, the amount of PI correlates with the DNA content, and accordingly, DNA fragmentation in an apoptotic cell translates into a sub G_0_–G_1_ peak (Sarkar et al., 2008). Promastigotes treated with AALEO caused cell cycle arrest at sub G_0_–G_1_ phase (**Figure 5**). The proportion of cells in the sub G_0_–G_1_ phase was 1.69% in the untreated control, which increased to 33.14 and 37.19%, respectively, in the promastigotes treated with AALEO and pentamidine. Thus, the data clearly show that AALEO induced cell cycle arrest at sub G_0_–G_1_ phase.

### AALEO INDUCES DEPOLARIZATION OF MITOCHONDRIAL MEMBRANE (Ψm)

**FIGURE 6:**
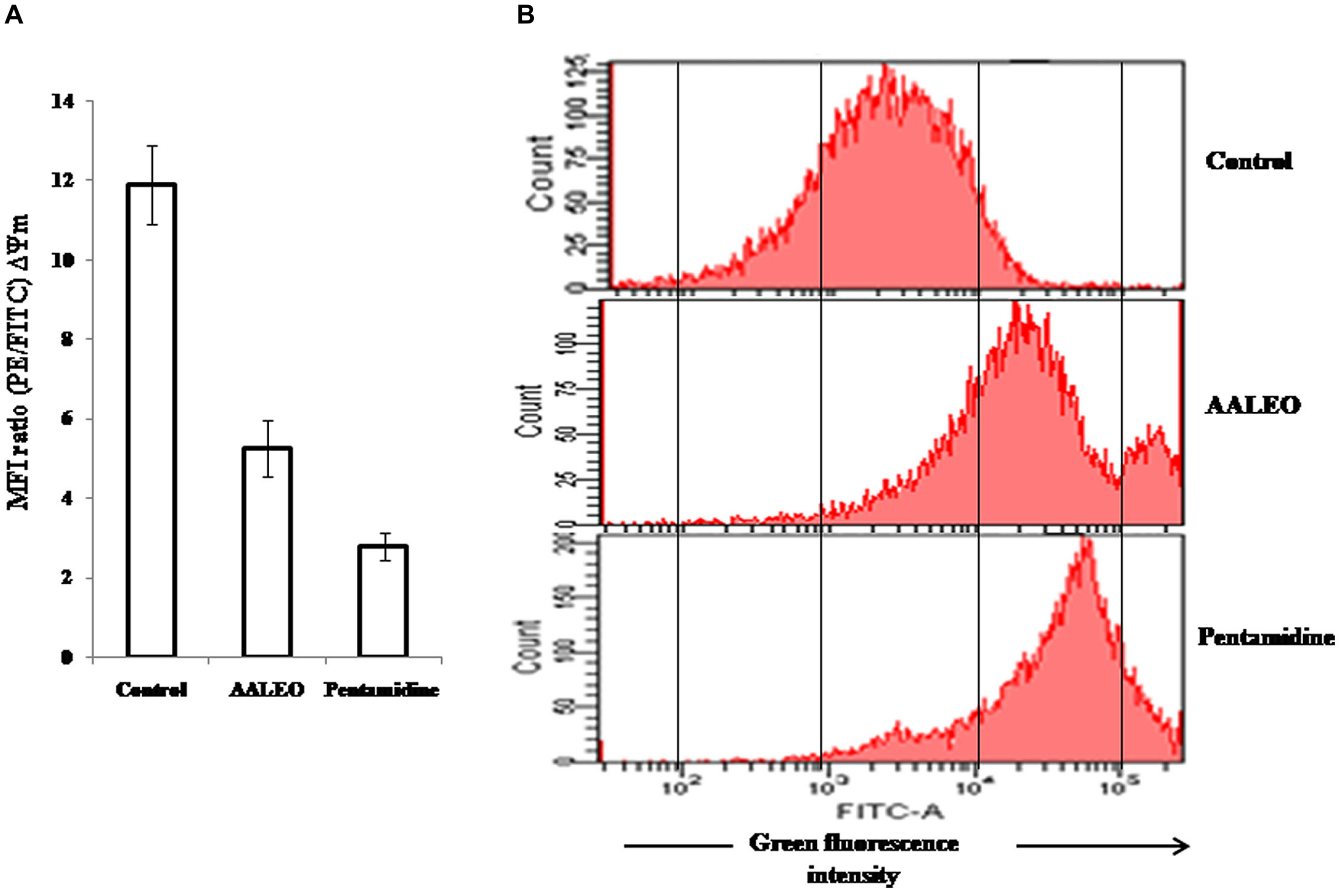
**Collapse of mitochondrial membrane potential and ROS generation in *L. donovani* promastigotes by AALEO.** Exponential phase promastigotes were incubated with AALEO and pentamidine (100 μg ml^-1^) for 72 h and **(A)** probed with JC-1; bar diagram represents the ratio of red/green fluorescence intensity (MFI) obtained from histogram statistics. **(B)** Histograms depict shift in green fluorescence intensity (FL-1) from left, depicting increased production of ROS in treated promastigotes loaded with H_2_DCFDA. Each bar corresponds to the mean ± SE of triplicate samples and histograms are representative profiles of three independent experiments.

Alteration of mitochondrial membrane potential (ΔΨm) is a characteristic feature of metazoan apoptosis and has been show to play a key role in drug-induced death in the unicellular protozoan parasites (Sen et al., 2007). The loss of ΔΨm was determined using a mito-sensor dye, JC-1. JC-1 is a cationic lipophilic fluorescent dye that freely permeates the mitochondrial membranes and forms J-aggregates in healthy cells, exhibiting pronounced red fluorescence. An apoptotic stimulus induces a decrease in the ΔΨm and JC-1 fails to enter the mitochondria, remaining as cytosolic monomers and emits green fluorescence. Therefore, the ratio of J-aggregates/monomers serve as an effective indicator of the mitochondrial energy state of the parasites, allowing apoptotic cells to be easily distinguished from their non-apoptotic counterparts (Verma et al., 2007). In control promastigotes, the red/green fluorescence ratio was 11.9. Treatment with AALEO (100 μg ml^-1^) and pentamidine for 72 h, induced a significant decrease in ΔΨm, resulting in a predominance of JC-1 monomers that fluoresced green, thus, translating into a decrease in the red/green fluorescence ratio to 5.27 and 2.79, respectively (**Figure 6A**).

### AALEO INDUCES PRO-OXIDANT ACTIVITY IN *L. donovani* PROMASTIGOTES

To evaluate the effect of AALEO on the oxidative status of promastigotes, H_2_DCFDA, a lipid soluble, membrane permeable compound was used that following cleavage by cellular non-specific esterases, forms an impermeable H_2_DCF, which is subsequently oxidized by intracellular ROS to produce a fluorescent compound, DCF. Therefore, the resultant fluorescence (green) is directly proportional to the quantum of ROS generated. AALEO (100 μg ml^-1^) treatment of promastigotes for 72 h led to significantly enhanced ROS levels with increase in the green fluorescence intensity (FI-1), in comparison to the control untreated parasites as observed by flow cytometry (**Figure 6B**).

### AALEO ACTIVITY AGAINST *L. donovani IN VIVO*

**FIGURE 7:**
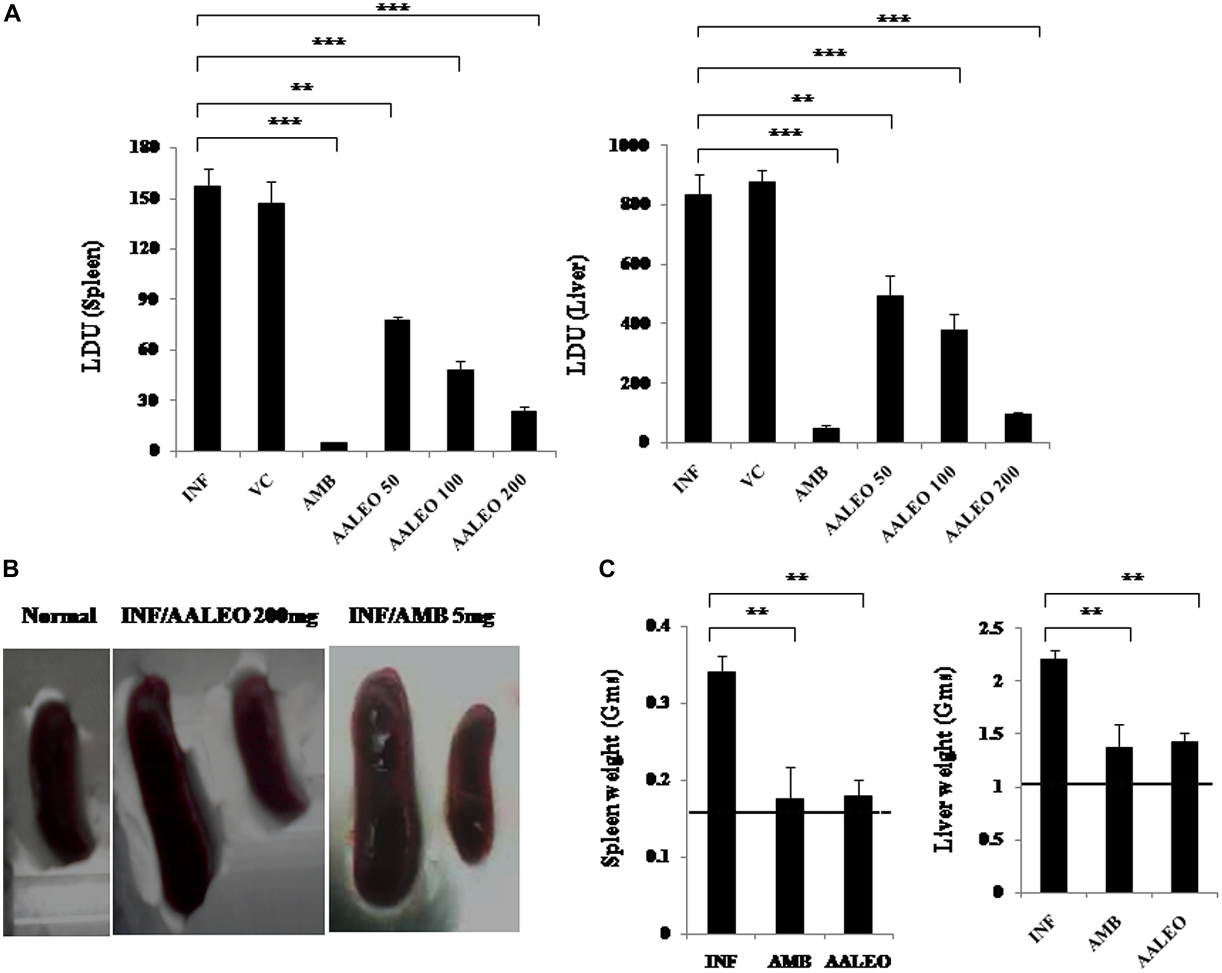
**Antileishmanial efficacy of AALEO on established *L. donovani* infection.** Ten weeks infected BALB/c mice were left untreated (INF) or treated for 10 days with PBS (VC), AALEO (50, 100, or 200 mg/kg bw, daily, i.p.) or amphotericin B (AMB; 5 mg/kg bw, alternate days, i.v.) as elaborated in Section “Materials and Methods.” Ten days post-treatment **(A)** splenic and hepatic parasite burden (LDU) were determined by stamp smear method, **(B)** morphology of spleen, and **(C)** weights of liver and spleen were also determined. The dotted lines indicate the respective organ weights of naïve mice. Data represent the mean ± SE for five animals per group. Data were tested by ANOVA. Differences between means were assessed for statistical significance by Tukey’s test (**P≤ 0.01; ***P≤ 0.001). Results are from one of three representative experiments.

Treatment of *L. donovani* infected BALB/c mice with AALEO at 50, 100, and 200 mg/kg b.w. resulted in 45.26 ± 8.95, 63.23 ± 7.28, and 88.68 ± 5.52 % parasite reduction, respectively, in the liver, while decrease in the splenic parasite load was more evident, being 72.48 ± 3.88, 80.72 ± 6.61, and 91.66 ± 3.07 %, respectively (**Figure 7A**). With AMB, parasite elimination in the liver and spleen was 94.02 ± 1.81 and 98.09 ± 2.44 %, respectively. Compared to the infected controls, a significant reduction in spleen and liver weight was also observed with AALEO (200 mg/kg b.w.) and AMB (**Figure 7C**). Morphological observation of spleen in the treated groups also demonstrated reduction in size that was comparable to the naïve group (**Figure 7B**).

### *IN VIVO* TOXICITY STUDIES

Estimation of serum SGOT, SGPT, and ALP for liver dysfunction and urea and creatinine for renal dysfunction, 10 days post injection of AALEO in normal BALB/c mice as well as infected and treated mice demonstrated normal levels of the serum enzymes. AALEO (up to 200 mg/kg b.w.) proved to be non-toxic in BALB/c mice used in antileishmanial screening (**Tables 2A,B**) indicating no *in vivo* toxicity.

**Table 2:** **(A)** Effect of AALEO on hepatic and renal functions of normal BALB/c mice. **(B)** Effect of AALEO on hepatic and renal functions of infected BALB/c mice post-treatment.

**(A)**
**Group (*n* = 5)**	**SGOT (U/L)**	**SGPT (U/L)**	**ALP (U/L)**	**Urea (mg/dL)**	**Creatinine (mg/dL)**

Control	52.40 ± 6.18	33.09 ± 10.06	86.14 ± 1.59	18.12 ± 3.17	0.96 ± 0.15
Essential oils (EO) 200 mg/kg (i.p)	53.16 ± 4.56	33.18 ± 4.36	87.18 ± 7.96	16.79 ± 4.91	0.84 ± 0.08

*Mice (*n* = 5) received EO daily for 10 consecutive days. Enzymatic estimations (mean ± SE) were done using commercial kits.*					

**(B)**
**Group (*n* = 5)**	**SGOT (U/L)**	**SGPT (U/L)**	**ALP (U/L)**	**Urea (mg/dL)**	**Creatinine (mg/dL)**

Infected control	48.40 ± 5.58	29.78 ± 3.77	89.42 ± 3.8	15.86 ± 2.60	1.16 ± 0.39
AmB 5 mg/kg (i.v)	53.93 ± 2.78	38.10 ± 1.16	85.36 ± 1.7	18.19 ± 1.90	0.99 ± 0.14
EO 50 mg/kg (i.p)	45.60 ± 5.39	29.99 ± 5.62	83.19 ± 2.9	16.24 ± 0.96	0.89 ± 0.11
EO 100 mg/kg (i.p)	42.43 ± 4.29	33.21 ± 3.07	87.31 ± 11.6	18.96 ± 1.93	1.00 ± 0.16
EO 200 mg/kg (i.p)	51.60 ± 0.56	31.78 ± 3.36	84.18 ± 5.9	17.49 ± 1.69	1.09 ± 0.10

*Mice (*n* = 5) received EO daily for 10 consecutive days. Enzyme estimations (mean ± SE) were done using commercial kits.*

## DISCUSSION

Natural products, especially EO from the aromatic herbs, have shown a wide spectrum of biological activities including antifungal (Kordali et al., 2008) antibacterial (Silva et al., 2010) and antiprotozoal (Machado et al., 2010) among others. Plant EO and active components can be used as alternatives or adjunct to current antiparasitic therapies. Leishmanicidal activity of EO has recently been documented (Rodrigues et al., 2013; Sanchez-Suarez et al., 2013) such as linalool-rich essential oil from leaves of *Croton cajucara* (Rosa et al., 2003), eugenol-rich essential oil from *Ocimum gratissimum* (Ueda-Nakamura et al., 2006) and sesquiterpenes-rich essential oil from *Eugenia uniflora* (Rodrigues et al., 2013) that have been shown to be cytotoxic against *L*. *amazonensis* parasites. EO from *A. herba-alba* and *A. annua* have been reported to exhibit antifungal, and antibacterial activities (Juteaua et al., 2002). In the present study, we reported antileishmanial activity of the essential oil from AALEO. The essential oil of *A. absinthium,* having oxygenated monoterpene camphor as the major constituent has been reported to exhibit activity against the promastigotes and amastigote forms of *L. aethiopica* and *L. donovani* (Tariko et al., 2011). Our findings are in agreement with these studies as the GC-MS analysis of AALEO revealed significantly higher content of camphor (52.06%) followed by β-caryophyllene (10.95%).

The camphor-rich essential oil of *A. annua* exhibited a dose-dependent significant antileishmanial effect against the promastigotes as well as the intracellular amastigotes of *L. donovani*. The effect was leishmanicidal as evidenced by growth reversibility analysis and alterations in cellular morphology including shrinkage in the promastigotes that became round in shape with disrupted flagella and had no motility.

The leishmanicidal activities of the plant secondary metabolites such as curcumin (Das et al., 2008), racemoside A (Dutta et al., 2007), artemisinin (Sen et al., 2007), *Aloe vera* leaf exudates (Dutta et al., 2008), and ethanolic extract of leaves of *Piper betle* (Sarkar et al., 2008) have been reported to be mediated by apoptosis. *Leishmania* parasites can undergo PCD in response to natural compounds and EO (Dutta et al., 2007; Sen et al., 2007; Das et al., 2008; Misra et al., 2009; Islamuddin et al., 2014). The mode of cell-death caused by AALEO was further investigated, since to the best of our knowledge, no such study on AALEO or camphor has been reported earlier.

During PCD in unicellular parasites, PS is externalized from the inner to the outer surface of the plasma membrane. In our studies, AALEO-treated *L. donovani* promastigotes also caused PS exposures at a significant rate as evidenced by the increased annexin V binding. The changes occurring in the nuclear material during PCD were further characterized. *In situ* TUNEL of nicked DNA was observed with AALEO thus, strongly substantiating apoptosis in *L. donovani* promastigotes. This has also been evidenced with essential oil from *Piper betle* and *Syzygium aromaticum* (Misra et al., 2009; Islamuddin et al., 2014).

DNA fragmentation in an apoptotic cell translates into a sub G_0_–G_1_ peak. Cell cycle analysis with PI showed a significant increase in the proportion of cells in the sub G_0_–G_1_ phase when treated with AALEO. This confirmed that the apoptotic cell was arrested at the sub G_0_–G_1_ phase. Similar observation has been reported with other natural antileishmanial molecules such as curcumin, artemisinin and berberine chloride, wherein, a substantial population of cells have been identified in the sub G_0_–G_1_ phase (Sen et al., 2007; Das et al., 2008; Saha et al., 2009; Islamuddin et al., 2014).

The mitochondria of the parasites seem to be central to the cytotoxic action of different natural and synthetic molecules such as pentamidine, miltefosine, and essential oil of *S. aromaticum* that have caused dyskinetoplastidy in *L. donovani* promastigotes (Verma et al., 2007; Islamuddin et al., 2014). In the present study, we found that the AALEO-induced leishmanicidal effect was accompanied by dyskinetoplastidy as evidenced by PI staining, corroborating the earlier studies.

Permeabilization of the outer and inner mitochondrial membranes can lead to cell death by apoptosis or necrosis. The loss of mitochondrial membrane potential (ΔΨm) is generally an early change associated with apoptosis as the dissipation of ΔΨm following permeabilization of the inner mitochondrial membrane triggers the release of several apoptotic factors. Artemisinin, berberine chloride, racemoside A, *Piper betle*, and essential oil of *S. aromaticum* have been reported to demonstrate leishmanicidal activity that was associated with a loss in ΔΨm (Dutta et al., 2007; Sen et al., 2007; Misra et al., 2009; Saha et al., 2009). In our studies, AALEO too caused mitochondrial membrane depolarization.

In multicellular and unicellular organisms, the mitochondria serve as an important cellular source for generation of ROS and reactive nitrogen species (RNS), critical for induction of apoptosis. The production of ROS during early phase of apoptosis usually follows an imbalance in the cellular redox homeostasis. Withaferin A, curcumin, ethanolic extracts of *Piper betle* and eugenol-rich essential oil from *S. aromaticum* (Dutta et al., 2007; Das et al., 2008; Misra et al., 2009; Islamuddin et al., 2014) have been reported to induce apoptosis by generating ROS in promastigotes. This prompted us to evaluate whether AALEO could elicit oxidative stress in *L. donovani*. In our study, we found that AALEO triggered the production of ROS within the promastigotes that might have contributed to their apoptotic death. NO intermediates have earlier been reported to intercede antileishmanial effect by *Kalanchoe pinnata* (Da-Silva et al., 1999). NO has also been reported to cause extensive fragmentation of nuclear DNA in both axenic and intracellular amastigotes of *L. amazonensis* (Holzmuller et al., 2002). Our studies are in agreement with the previous reports as a significant increase in NO was observed after exposure of amastigote-infected macrophages to AALEO. Generation of NO following drug treatment in infected macrophages further indicated the involvement of RNS in amastigote death. EO from *Ocimum gratissimum* (Ueda-Nakamura et al., 2006) and *Croton cajucara* (Do Socorro et al., 2003) have shown similar NO enhancing activities.

We further explored the therapeutic efficacy of AALEO against VL using *L. donovani* infected BALB/c mice. The major findings emerging from this study are that AALEO (200 mg/kg b.w) resulted in maximum clearance of parasites (88–91%) from the liver and spleen of mice with established *L. donovani* infection as compared to untreated infected controls. Significant reduction in spleen and liver weight was also observed with AALEO. Similar therapeutic effect has been reported with the essential oil of *Chenopodium ambrosioides* (Monzote et al., 2006) and *Bixa orellana* (Monzote et al., 2013).

The hepatic and renal toxicities in normal and *L. donovani* infected BALB/c mice upon subsequent administration of AALEO (upto 200 mg/kg b.w.) were evaluated by monitoring the serum levels of SGOT, SGPT, ALP, urea, and creatinine. The enzymatic levels were in the normal range, indicating preclusion of hepato- and nephro-toxicity. In *ex vivo* studies also, AALEO was not found to compromise the macrophage viability.

**FIGURE 8:**
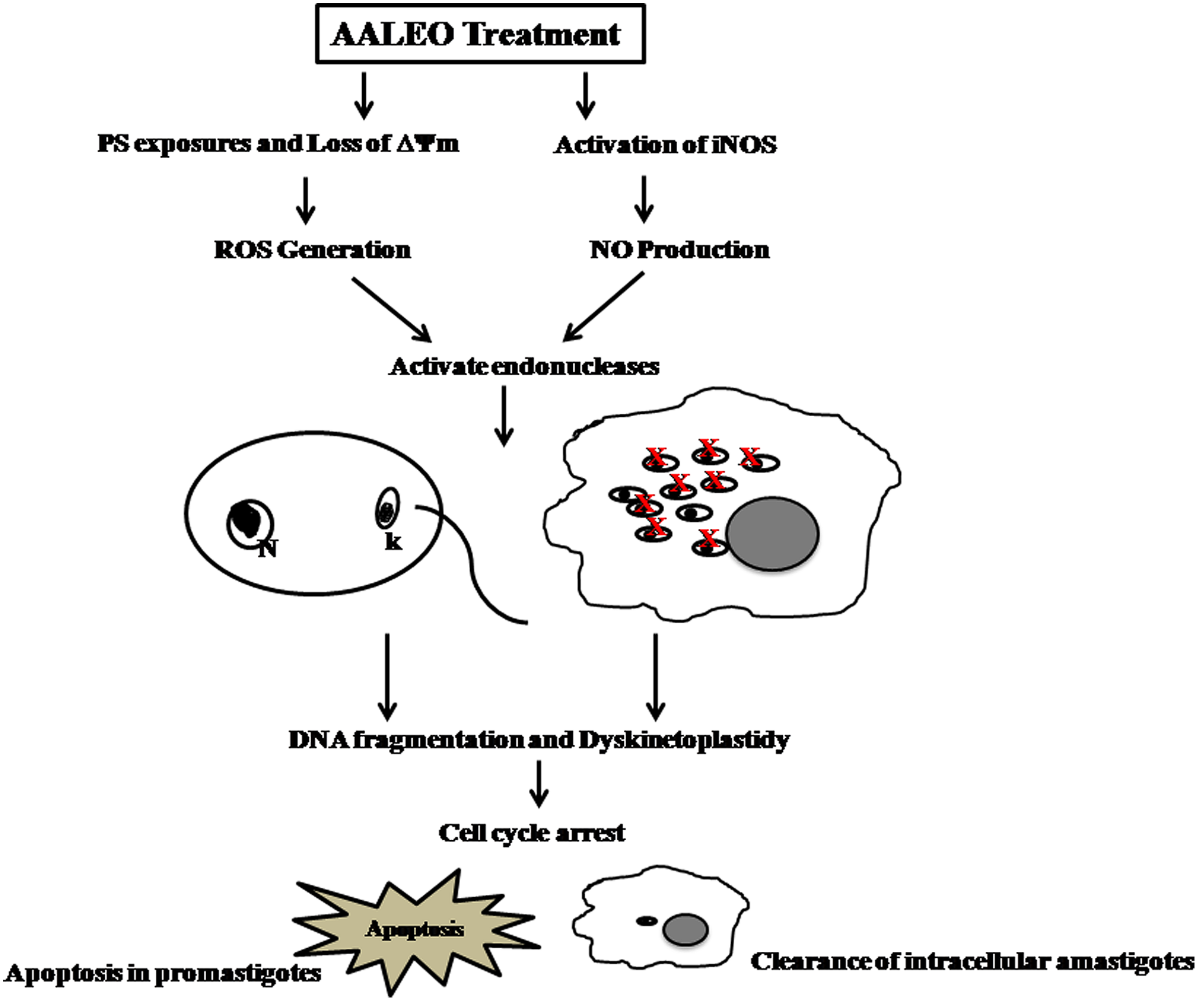
**Proposed model for AALEO-induced PCD in *L. donovani* promastigotes and amastigotes**.

Thus, we conclusively demonstrate that camphor-rich oil of AALEO exhibited antileishmanial efficacy against the promastigotes and intracellular amastigotes. The leishmanicidal activity was further confirmed in *L. donovani* infected BALB/c mice where ≥90% inhibition of parasite burden was observed. The leishmanicidal effect was mediated by PCD as evidenced by PS externalization, *in situ* DNA nicking, dyskinetoplastidy, cell cycle arrest at sub- G_0_–G_1_ phase, reduction of ΔΨm, ROS, and RNI generation (**Figure 8**). Moreover, no cytotoxic effect was observed on the mammalian macrophages and there was no impairment of liver and kidney functions of BALB/c mice treated with AALEO. Our study widens the scope for future designing and strengthening of the chemotherapeutic strategies for better management of this debilitating disease.

## AUTHOR CONTRIBUTIONS

MI, FA conceived and designed the experiments. MI, FA, GC performed the experiments. MW assisted in *ex vivo* experiments while MT, DS, and MA assisted in extraction of oil. FA, MI analyzed the data and wrote the manuscript.

## Conflict of Interest Statement

The authors declare that the research was conducted in the absence of any commercial or financial relationships that could be construed as a potential conflict of interest.
