# Corrigendum: Leishmanicidal activities of *Artemisia annua* leaf essential oil against Visceral Leishmaniasis

**DOI:** 10.3389/fmicb.2015.01015

**Published:** 2015-09-24

**Authors:** Mohammad Islamuddin, Garima Chouhan, Muzamil Y. Want, Maujiram Tyagi, Malik Z. Abdin, Dinkar Sahal, Farhat Afrin

**Affiliations:** ^1^Parasite Immunology Lab, Department of Biotechnology, Jamia Hamdard (Hamdard University)New Delhi, India; ^2^Department of Biotechnology, Centre for Transgenic Plant Development, Jamia Hamdard (Hamdard University)New Delhi, India; ^3^Malaria Group, International Centre for Genetic Engineering and BiotechnologyNew Delhi, India; ^4^Department of Medical Laboratories Technology, Faculty of Applied Medical Sciences, Taibah UniversityMedina, Saudi Arabia

**Keywords:** leishmaniasis, visceral, essential oil, *Artemisia annua*, leishmanicidal, apoptosis, therapeutic efficacy

The author Muzamil Y. Want was inadvertently missed in the original manuscript and we wish to add his name for contribution to *ex vivo* experiments.

## Author contributions

MI, FA conceived and designed the experiments. MI, FA, GC performed the experiments. MW assisted in *ex vivo* experiments while MT, DS, and MA assisted in extraction of oil. FA, MI analyzed the data and wrote the manuscript.

The affiliation/correspondence address of the Corresponding author had a minor error, which we hereby rectify. It was incorrectly written as:

Department of Medical Laboratories Technology, Faculty of Applied Sciences, Taibah University, PO Box 344, Universities Road, Medina 30001, Saudi Arabia

The correct version is:

Department of Medical Laboratories Technology, Faculty of Applied Medical Sciences, Taibah University, PO Box 344, Universities Road, Medina 30001, Saudi Arabia

### Conflict of interest statement

The authors declare that the research was conducted in the absence of any commercial or financial relationships that could be construed as a potential conflict of interest.

